# Editorial: Fungal Bioactive Metabolites of Pharmacological Relevance

**DOI:** 10.3389/fphar.2022.912068

**Published:** 2022-06-08

**Authors:** Pius S. Fasinu, Festus B. C. Okoye, Oyindamola O. Abiodun, Ramsay S. T. Kamdem, Omonike O. Ogbole

**Affiliations:** ^1^ Department of Pharmacology and Toxicology, University of Alabama at Birmingham, Birmingham, AL, United States; ^2^ Department of Pharmaceutical and Medicinal Chemistry, Faculty of Pharmaceutical Sciences, Nnamdi Azikiwe University Awka, Awka, Nigeria; ^3^ Department of Pharmacology and Therapeutics, University of Ibadan, Ibadan, Nigeria; ^4^ Institut für Organische and Analytische Chemie, Universität Bremen, Bremen, Germany; ^5^ Department of Pharmacognosy, Faculty of Pharmacy, University of Ibadan, Ibadan, Nigeria

**Keywords:** bioactive metabolites, drugdiscovery, endophytic fungi, fungal pharmacology, fungal secondary metabolites

## Introduction

The eukaryotic organisms that constitute the kingdom fungi have, for a long time, been of pharmaceutical and industrial interest as they serve as natural sources of important bioactive secondary metabolites. Compared to other plant-derived active products, biochemicals from fungi represent an underexplored source despite the historical contribution of this kingdom to the discovery of antibiotics (penicillin, cephalosporins, among others), immunosuppressants (including cyclosporin, mycophenolic acid), antifungal drugs (griseofulvin, echinocandins), cholesterol-lowering statins, anti-migraine ergot alkaloids, among others. In spite of being proven sources of pharmaceuticals, only a fraction of the described species of fungi has been screened for drugs. Most fungi studies are focused on the late-diverging fungal phyla (Ascomycota and Basidiomycota), while other phyla remain mostly un-investigated.

Edible and medicinal mushrooms are one of the most widely studied fungi for their biologically active compounds. Another group that has been of interest is the endophytic fungi, particularly those that grow in marine habitats. Recent studies have reported bioactive compounds from these endophytic fungi (Chávez et al., 2015). A unique property of this class of fungi is that they are amenable to manipulation at a laboratory scale and their production of unique secondary metabolites can be enhanced by various modulating techniques like media engineering, co-culture, chemical induction, epigenetics and metabolite remodelling.

In this current edition, Begum et al. explored the potential antidiabetic activity of the phytochemical constituents of *Morchella conica*. *M. conica* is a popular wild edible mushroom in parts of Asia and Europe. By targeting the Protein tyrosine phosphatase 1B (PTP1B), an important regulator in the insulin signalling pathways associated with insulin resistance, the authors set out to, not only seek a new potential antidiabetic drug source, but also a unique target for which no commercial antidiabetic drug currently exists. Compared to controls, extracts of *M. conica* potently inhibited PTP1B *in vitro*, while significantly decreasing blood sugar levels in diabetic-model mice. These results were accompanied by positive markers of improved renal and hepatic functions. Expanding the frontiers of diabetic treatment through novel biomolecular mechanisms that counter insulin resistance is a worthwhile adventure. The potential of bioactive secondary metabolites from fungi to achieve this feat is worth further research.

Another endophytic fungus that features in this special edition is *Aspergillus terreus*. In the study conducted by Sakaida et al., a bioactive secondary metabolite isolated from *A. terreus* (+)-terrein demonstrated *in vitro* suppressive activity against the phosphorylation of PKCα/βII that is typically induced by the receptor activator of nuclear factor-κB ligand (RANKL). RANKL plays important role in bone resorption and the suppression of its activity has been one of the core therapeutic targets for the prevention and management of osteoporosis. For example, denosumab, an approved drug for the treatment of osteoporosis, works by suppressing osteoclastic activities through the inhibition of RANKL. As a monoclonal antibody, denosumab is not just the only approved drug in the class, it is associated with a wide range of adverse effects including immunosuppression and increased risk for systemic infection. However, RANKL inhibition is still one of the pharmacologically prudent ways of preventing and treating osteoporosis compared to bisphosphonates-mediated osteoclastic inhibition. Sakaida et al. have shown that (+)-terrein modulates different processes including the suppression of the nuclear factor of activated T-cell 1 (NFATc1) which regulates osteoclastogenesis. In translating these *in vitro* biomodulation to potential anti-osteoporotic activity, the authors demonstrated that the administration of (+)-terrein in mice significantly improved bone density, bone mass, and trabecular number.

From this study by Sakaida et al., *A. terreus* as a source of a bioactive compound highlights the continuing discovery of new chemical compounds with potential therapeutic and pharmaceutic utility. While the current study focuses on the potential benefit in osteoporosis (+)-terrein has been evaluated for several other potential utilities including cancer and inflammatory diseases.

The study by An et al. demonstrates the continuing ability of various fungi to produce secondary metabolites through the exploration of endophytic relationship with other plants. The study showed the biotransformational ability of *Panax bipinnatifidus*, from where the authors isolated 93 representative morphotype strains of fungi, out of which a 26 exhibited antibacterial/antifungal activity. The enormity of the potential of fungi to continue to serve as sources of antibiotics is still underappreciated. While bacterial resistance to the existing antibiotics has been a concern in pharmacotherapy, the evolutionary ability of the fungi to manufacture bioactive substances of varying and unrelated chemical structural backgrounds can continue to offer hope against infectious diseases.

Ye et al. explored the chemistry and biodiversity of the endophytic fungus *Ilyonectria robusta*. This study reported the isolation and characterization of five new isopimarane diterpenoids possessing the unique skeletons of 19-nor-isopimarane or isopimarane. Two of the new compounds, robustaditerpene C and robustaditerpene E exhibited immunosuppressive activity against cell proliferation of B and T lymphocytes respectively. The recent applications of compounds with immunomodulatory potentials in the management of emerging disease states further underscore the potentials of endophytic fungi in generating novel, sustainable and potent bioactive “lead” molecules.

Furthermore, metabolomic and transcriptomic changes in the sclerotia of *Polyporus umbellatus* as a result of the symbiotic relationship with *Armillaria mellea* were reported by Mei Xing et al. in this special edition. *Armillaria melea,* a saprophytic fungus with facultative and parasitic activity is capable of infecting and establishing a symbiotic relationship with *Polyporus umbellatus*. This relationship plays critical role during the formation and development of the sclerotia, the part of *P. umbellatus* with medicinal properties. Metabolomic and transcriptomic techniques were used to determine the difference between metabolites found in the infected and uninfected parts of the separated sclerotia cavity wall of *A. melea*. A total of 118 metabolites between the two groups were differentially expressed and identified. The pathways of metabolism suggested that carbohydrates, fatty acids as well as steroids were highly enriched and active during the infection of the sclerotia part of *P. umbellatus* by *A. melea*. In comparison to that of the control group, the content of polyporusterone A and B, and that of ergosterol in *A. melea* infected *P. umbellatus* were increased by 75.0%, 20.0%, and 32.2%. Series of enzymes that played significant functions in the last steps of the biosynthesis of ergosterol, were upregulated in *A. melea* infected *P. umbellatus*. It appears, that *A. melea* drives the modification of the chemical components of *P. umbellatus* sclerotia.

During the past decades, several novel compounds with diverse biological activities have been identified and isolated from fungi. As a kingdom that comprises over 1.5 million species, with only a few thousand explored so far, fungi still represent a vastly untapped reservoir of chemical backbones and actual bioactive for potential therapeutic uses. The diversity in the ecology, morphology and physiology of fungi makes them more scientifically vital for pharmaceutical research. One of the major challenges in this regard is the reliance on only culturable and fast-growing fungi for drug screening. The application of genetic engineering for cloning unculturable fungi into heterologous fast-growing hosts can expand the exploratory opportunities in fungi-sourced drug discovery research.
